# Conscience at the End of Life

**DOI:** 10.3390/nursrep14040298

**Published:** 2024-12-19

**Authors:** Ralph Neil Baergen, James Skidmore

**Affiliations:** Department of Philosophy, Idaho State University, Pocatello, ID 83209, USA; ralphbaergen@isu.edu

**Keywords:** medical ethics, conscientious refusal, conscientious commitment, end of life, nursing, healthcare law

## Abstract

Background/Objectives: Caring for patients at the end of life can involve issues that are ethically and legally fraught: withholding or withdrawing artificial nutrition and hydration, pain control that could hasten death, aggressive treatment that is continued when it seems only to be prolonging suffering, patients who request medical assistance in dying, and so forth. Clinicians may find that their deeply held ethical principles conflict with law, institutional policy, or patients’ choices. In these situations, they may consider either refusing to participate in procedures that they find morally abhorrent (conscientious refusal) or providing care that they believe to be ethically obligatory despite being contrary to law or policy (conscientious commitment). Methods: This paper reviews the ethical issues involved. Results: Each of the usual policies for handling conscientious refusals faces serious challenges. Conclusions: Healthcare providers who refuse to provide medical services should be expected to explain their reasons, make prompt referrals, and bear some of the resulting costs or burdens.

## 1. Introduction

Appeals to conscience are attempts to receive exceptions from involvement in medical procedures or compliance with laws or policies that one finds morally objectionable or religiously impermissible. Those who make such conscientious objections commonly believe that they should be accommodated without penalty [1]. Initially, conscientious refusals involved abortion, but in the past few decades, they have spread to other areas of medical practice, particularly situations near the end of life.

Conscientious refusals differ from civil disobedience. Civil disobedience is the overt, public violation of a law in order to draw the public’s attention and pressure authorities to change a law one regards as unjust; someone engaged in civil disobedience typically expects to be punished. Conscientious refusal, by contrast, is not per se intended to change law or policy and need not involve any expectation of punishment or willingness to accept such punishment. Nor does conscientious refusal per se involve obstructing others from providing the medical service one finds objectionable; in its purest form, conscientious refusal is about avoiding moral wrongdoing, not protecting the patient’s moral character [2].

Ethics is central to the health professions. The practice of medicine is all about doing good and helping people, and clinicians often see their professional roles as woven into their identity—they are people who are committed to doing good. In addition to technical competence and knowledge, healthcare professionals are expected to have ethical competence to identify and thoughtfully navigate the issues that arise in patient care. Obviously, patients also have a morally important interest in how healthcare is delivered, too. When a democratic society decides that a particular medical service is legal, patients are entitled to have access to it when it is medically appropriate. If a healthcare provider refuses for reasons of conscience, that can affect the patient’s basic rights and liberties, often involving bodily integrity, suffering, life, and death [3].

Clinicians are not simply healthcare vending machines to be manipulated by patients and employers. Rather, they are moral agents, accountable for their actions and guided by their own ethical standards. It is inevitable that an individual’s moral judgments will sometimes lead them to choices that conflict with what patients or their families have chosen, or with what law and institutional policy require of them. In such situations, each healthcare professional must decide whether to act in violation of their moral judgment or to act in ways contrary to what patients, employers, and society expect of them.

## 2. Refusals at the End of Life

Much of the discussion of conscientious refusal has focused on abortion and reproductive technologies, but many situations near the end of life are relevant as well:

- Providing treatment clinicians believe to be futile or that merely prolongs dying (including attempted resuscitation) [4];
- Organ donation after the circulatory determination of death;
- Withholding or withdrawing medically provided nutrition and hydration;
- Terminal sedation (continuous deep sedation at the end of life) [5];
- Medically assisted suicide or euthanasia [5,6].

A physician may conscientiously refuse to act on a patient’s medical choices when doing so would bring about death prematurely or unnecessarily.

*Case: spinal injury.* A spinal injury leaves a patient paralyzed and respirator-dependent. One week post-injury, he is conscious, alert, and deeply unhappy with his situation. He has not yet had time to understand the details of his injury or learned about the supports and assistance available; he has never met anyone living with this level of disability. The patient insists that he wants the ventilator turned off so that he can die. His only close relative is a brother who supports this choice and threatens to sue if it is not respected. The doctor refuses, saying that it is much too soon to make that decision.

Even when death is imminent and inevitable, a clinician’s conscience may rebel at doing anything that would hasten that death or make them causally responsible for it.

*Case*: *neuromuscular paralysis*. A patient with end-stage COPD has been intubated and put on a ventilator, but this has not brought about enough improvement. The patient and family understand that he may not survive this hospitalization, and the patient is adamant that he does not want to be stuck on a ventilator for any longer than necessary. The family agrees to a trial of neuromuscular paralysis because there is a chance that, by improving tidal volumes, it might get him through this crisis. The family and physician agree at the outset that, if the patient does not show clear improvement by a specified date, then the treatment will be halted, and he will be terminally weaned from the ventilator. When that deadline arrives, his condition has not improved, and the family asks that their loved one be disconnected and allowed to die, as agreed. Although the effects of the paralytic drug can quickly be reversed, it takes longer for the patient’s muscles to recover so that he can make significant respiratory effort. The physician was willing to terminally wean if the cause of death was that the patient’s lungs were too damaged to keep him alive (even with the patient’s respiratory effort). The physician was not willing to do this if the patient was unable to make that respiratory effort (because muscles had not adequately recovered from paralysis) [7].

Not all refusals are rooted in moral judgments about the medical intervention itself. In some cases, it arises from the conviction that the intervention is contrary to the patient’s wishes or best interest; this often involves worries about futility. The most common sort of refusal is to direct participation in providing a medical service one regards as seriously morally wrong, but refusals are also seen in more peripheral forms of involvement, such as refusal to refer the patient to a colleague and even refusal to provide patients with information about treatment options the clinician regards as wrong [8]. Medical staff may refuse to schedule appointments, order supplies, prep a treatment space, or clean instruments used in a medical service to which they object. Conscience might also lead one to reduce one’s level of participation in something one finds morally objectionable without explicitly refusing. “Slow codes”, for instance, can be thought of as a borderline case of conscientious refusal to participate in a resuscitative effort one believes to be futile or contrary to the patient’s interests [9]. Rather than refusing to take part in the resuscitation effort at all, one does so in a manner that reduces the likelihood that it would be effective. This is more troubling than having an open discussion of the situation.

There are few current cases of conscientious refusal that are uncontroversially ethically justified; such refusals would challenge current standards of practice and so would typically be controversial. A healthcare provider’s moral justification for refusal will often be questionable, but cases can also arise in which a caregiver would be *right* to refuse.

*Case: degenerative brain disease.* A patient has oliviopontocereballar atrophy (OPCA), a rare degenerative brain disease for which there is no effective treatment. She is profoundly mentally disabled and unable to speak, read, walk, or eat. She has painful flexure contractions of all four limbs and is fed by means of a gastrostomy tube. At age 42, she was hospitalized for gastrointestinal bleeding that was initially diagnosed as aspiration pneumonia. She could no longer tolerate her G tube, had an “acute” abdomen, and was in severe abdominal pain. Her doctors agreed that further treatment would probably just prolong her suffering. Following meetings with the family, the hospital’s clinical ethics committee, and members of the clergy, it was agreed that she would receive palliative care only and that nutrition and hydration would be stopped. However, state law prohibited withholding nutrition, hydration, or antibiotics from someone with this patient’s cognitive disabilities, and a judge ordered that these be resumed. Attempts to provide 900 calories/day resulted in projectile vomiting and intractable hiccups. The patient was able to tolerate only 300 cal/day of IV fluids; this continued for 2.5 months. During this time, she developed organ damage, severe edema, and intractable pain. Eventually, the judge consented to stopping the IV and returning to palliative care only, even though this was contrary to state law. This order was immediately appealed, resulting in the reinstatement of IV and antibiotics. The patient died before that appeal could be heard. State law was subsequently changed to permit a patient’s guardian to choose palliative care only.

The treatment provided in this case was legally required despite being unethical. There is no record of any clinician involved in this patient’s care invoking conscience to refuse to carry out the court’s orders, but perhaps they should have. At the very least, they would have been morally justified in refusing [10].

## 3. Responding to Refusals

When a healthcare professional conscientiously refuses to provide a medical service, how should institutions or legal authorities respond? Proposals in the literature cover a wide range of positions [11]; these are summarized in Table 1. Some would refuse to accommodate any refusals, while others would accommodate (virtually) all of them. Most are somewhere in the middle, proposing criteria that a refusal must meet in order for it to be accommodated. (To accommodate a refusal means to allow the refuser to be exempt from participating in the medical service at issue. It may or may not involve having the patient receive that service from another provider.)

### 3.1. Override All Refusals

There are those who argue that healthcare professionals’ conscientious refusals should rarely or never be accommodated. Julian Savulescu is a notable example; he defends the position that no conscientious refusals should be permitted and that “doctor’s conscience has little place in the delivery of modern medical care” [12] (p. 294). In his view, healthcare providers are charged with the responsibility of delivering services competently, reliably, and without introducing their own views.

Overriding all conscientious refusals has the advantage of providing uninterrupted care to patients. The availability of medical services would be established by law and policy, as well as the vagaries of resource availability; the personal views of healthcare providers would never prevent patients from getting the care they want. However, this would require compelling some of those providers to act contrary to deeply held views about what is right and wrong.

Moral distress is experienced when one acts contrary to one’s conscience or when one knows the right thing to do but is unable to do it [13]. This is a substantial contributor to burnout and to healthcare professionals moving to different positions or leaving the profession altogether [14,15]. Moral distress is elevated for those who are dealing with families who insist on continuing treatment when it is not in the patient’s best interest and providing care that seems merely to cause suffering or prolong dying [16].

So, what should the conscientious healthcare provider do when asked to do something they believe is profoundly wrong? According to Savalescu, Cantor, and others, the answer is that typically one should push aside one’s moral qualms and get on with it [12]. “Conscience is a burden that belongs to the individual professional; patients should not have to shoulder it” [17] (p. 1485). The primary concern is not the clinician’s mental or moral state but the patient’s need to receive appropriate care without having to worry about whether it will be unexpectedly withheld or delayed, or whether the clinician will refuse to provide important information or make suitable referrals. When a conscientious refusal arises, “…patients have no notice, no process, and no advocate to whom they can turn” [17] (p. 1485).

Those who object to accommodating conscientious refusals tend to claim that the consequences for clinicians are trivial or, at least, insufficient to justify the disruptions refusals cause. Rhodes, for instance, is opposed to accommodating conscientious refusals and says, “The doctor who chooses to avoid personal psychic distress declares his willingness to impose burdens … on his patients so that he might feel pure” [18] (p. 7). Although some claims about the harm to clinicians seem overwrought (e.g., the claim that acting contrary to one’s conscience is “worse than death” [19] (pp. 17–18)), they cannot be dismissed entirely. Just what harm they experience would seem to vary case by case, person by person.

Moral stress is inevitable in healthcare. Clinicians face a daily parade of patients whose needs they are unable to meet, the consequences of unjust distribution of resources, and so on. It is important to distinguish between stress arising from these repeated lower-level breaches of conscience and that resulting from one (or a few) major clashes. Refusals that arise from deeply held moral principles, ones that are part of defining who you are as a moral being, should be given greater weight because overriding them will result in greater harm to the clinician [20].

If all conscientious refusals are rejected, there is a risk that we will unnecessarily continue with practices that are seriously unethical. Perhaps above all, it is important to remember that there can be morally justified case of conscientious refusal. The case of the patient with degnenerative brain disease, discussed above, is one example. Rejecting refusals in these cases involves forcing a caregiver to do what she rightly believes is morally wrong. Furthermore, medical history provides us with too many instances of medical practices that were widely accepted at the time but that we now regard as clearly wrong:

- Between 1907 and 1963, more than 60,000 people—those judged to be “feebleminded” or in other ways “degenerate”—were involuntarily sterilized in the U.S. The practice was legal, at one time or another, in 33 states [21].
- Until the 1960s, it was common for physicians to conduct medical experiments using human subjects without their informed consent. Subjects often suffered significant harm, including serious illness or death [22,23].
- Until 1974, homosexuality was diagnosed as a mental illness and treated using hormone therapy, electroconvulsive therapy, aversion therapy, lobotomy, and castration [24].

Prior to the patients’ rights movement of the 1960s, the paternalistic “doctor knows best” approach was dominant in medical care: it was assumed that physicians would provide the treatments they thought best as guided by their individual medical judgment and moral principles. Conscientious refusals first rose to prominence in Western medical settings in the 1960s and 1970s, usually involving abortion following its legalization in the United States and the United Kingdom. In the United States, the 1973 U.S. Supreme Court decision in *Roe v. Wade* was followed that same year by the Church Amendment (42 U.S.C. § 300a-7[b]), which protected clinicians’ refusals to be involved in abortion. From abortion, moral concern and conscientious objection soon spread to other reproductive technologies, as well as to the many ways in which patients could be kept alive far longer than before. Canada’s recent legalization of medical assistance in dying (MAID) has brought with it significant numbers of physicians and nurses who refuse to offer this service or participate in it [25].

As of this writing, at least 27 countries have laws protecting (to varying extents) HCPs who conscientiously object to abortion or other medical services [26]. These laws clearly have influenced the accessibility of some medical services. For instance, in 1991, 83% of US counties did not have an abortion provider [27].

Human beings are not finished making mistakes; there will be more instances in which a healthcare provider recognizes that something we are accustomed to doing is in fact morally wrong. We need to find the right balance: by making conscientious refusals hard enough that they are not done lightly, we minimize disruption to medical care but still make it possible to identify and call out serious moral wrongs.

### 3.2. Accommodate All Refusals

At the other extreme, there are those who argue that it is wrong to compel people to do what their conscience tells them they must not do; therefore, we have an obligation to accommodate virtually all healthcare providers’ conscientious refusals [28]. Those who refuse would not be obliged to explain or defend their reasons for doing so, and they would not be subject to punishment or other burdens for refusing. The burden of proof would be on patients and healthcare institutions to demonstrate that accommodating the refusal would be unacceptably burdensome. While this approach has the advantage of protecting refusers from moral distress and reducing burnout, patients will face very serious disruptions.

When you decide what to do in a situation, you are also deciding what sort of person you are (or want to be). If you choose to participate in something your conscience tells you is seriously wrong, you are choosing to be a bad person [29]. We should want healthcare professionals to be ethical, honorable people; this will have a profound influence on how they interact with patients. We ought, then, to be wary of pressuring them into acting contrary to their consciences; their conscientious refusals should be given considerable weight.

If we compel people to violate their core moral commitments, this violation may trigger a broader decline in moral character [2]. This would be particularly concerning in healthcare, where we expect professionals to have the moral competence to navigate complex situations. Appeals to conscience by healthcare providers show the diversity of our moral views and their divergent judgments about how providers ought to conduct themselves [30].

Healthcare professionals are allowed to consider their own interests in some ways. This is why it is permissible for them to take vacations, to refuse to make house calls, to avoid taking large financial losses, to specialize in a field of medicine they find interesting and rewarding, and so forth. Perhaps we should also accept that they can also consider their own interest in avoiding moral distress; this could add weight to the claim that conscientious refusals should be accommodated [2].

The ethical principle of respect for autonomy has been applied chiefly to patients, emphasizing their role in making treatment decisions. But it also applies to the clinicians providing that treatment. When an autonomous healthcare professional conscientiously refuses to provide a particular medical service, respect for autonomy obligates us to take that seriously; it must be given due weight in crafting our response. The clinician’s autonomy is, of course, only one part of the story; conscientious objections must always give due consideration to how a refusal would affect others. Theologian Dietrich Bonhoeffer wrote that invoking conscience to exempt oneself from involvement without considering how that refusal would affect others is morally empty self-justification [31], cited in [32].

Accommodating all conscientious refusals could open the door to abuses. One commonly expressed concern is that some healthcare professionals will use conscientious refusals to deny treatment to patients for their religions, gender identity, ethnicity, immigration status, and so forth. Laws passed in Florida and some other states explicitly withhold legal protection from those who refuse because of the patient’s race, ethnicity, color, religion, sex, or national origin, but nothing is said about refusing because of gender identity or marital status. Also, appeals to conscience are sometimes used as a cover for a desire to punish or avoid a patient.

*Case: punishing a patient*. Several patients are brought to the hospital emergency department from the scene of a motor vehicle collision. The occupants of one vehicle, a couple in their 30s and their two children, were seriously injured when the occupant of the other vehicle, a male in his 60s, failed to stop at an intersection and struck them. Of the family members, one child was declared dead at the scene, and the others have various life-threatening (and life-changing) injuries. The driver of the other vehicle is recognized by hospital staff as a “frequent flier” in their emergency department; he is often there with medical problems arising from his behavior while drunk (injuries from fights, frostbite from losing consciousness outdoors, etc.). He is drunk again, and his injuries are less serious; they include a dislocated shoulder and a fractured leg. He is conscious, in pain, and cursing at the medical team trying to treat him. A nurse is instructed to administer medication to ease his pain, but she refuses. She says that he needs to suffer for all the harm he has done.

When conscience is invoked as a reason for refusing to provide medical services to someone because of their ethnicity, color, substance use disorder, gender identity, immigration status, behavior, or other characteristics of the individual patient, it is often camouflage for bias [33]. It does not merit protection or accommodation.

Accommodating all conscientious refusals is presented as a way of respecting diverse views; some people’s conceptions of morality cannot be accepted. There can be no place in society for views that permit (or require) human sacrifice, slavery, and so on. Once those have been ruled out, there remains a good deal of space within the parameters set by John Stuart Mill’s view of liberty—roughly, the principle that my freedom to swing my fist stops where your nose begins [34]. National and state authorities should not promote any particular view of the good but should permit citizens to formulate and pursue their own views (within the usual limits). This implies that we should do what we can to accommodate healthcare professionals’ conscience claims [3].

When a patient’s medical care is interrupted by someone’s conscientious refusal, there are typically delays and perhaps additional costs as well. Disease may progress, risk may increase, suffering must be endured, and medical bills add up. If the patient has autonomously chosen the care that is being refused, then respect for that person’s autonomy is not being upheld. The burden of refusals does not fall equally on all patients. Those who live in urban areas are more likely to be near other healthcare providers who would be willing to treat them, and those who are better insured or financially better off will be better able to handle the costs involved. But people in marginalized groups, those in rural areas, those living in poverty, those who have inadequate insurance, and so on are likely to be hit harder by a clinician’s refusal [3]. Patients and families are affected by refusals in ways that go beyond delay or disruption of care. They may also feel they are being stigmatized and judged as immoral—particularly because this comes from someone with social status and power. For instance, a physician in Missouri refused to provide HIV prophylaxis to a bisexual patient because it would “enable immoral sexual behavior” [1]. This would be magnified for patients who had developed trusting relationships with their healthcare providers.

Although conscience is important, it is not (as we shall see) infallible. LaFollette puts it well: [35] (p. 45)

“Nonetheless, although I argue that this absolutist claim of a right to conscience is indefensible, the advocates’ rhetoric expresses a hope most of us share. We would like to live in a world where we are never required to act in ways we think are immoral; we would like never having to suffer because of our moral choices. Unfortunately, this hope is a fantasy. Avoiding doing what we deem wrong sometimes comes at a considerable cost. This is not a problem unique to professionals. It is a fact of work, life, and morality”.

#### 3.2.1. Conscientious Refusal Law

We are not aware of any philosopher or medical ethicist who says that conscience is an infallible guide to moral judgment and that it must never be overridden. There are, however, some lawmakers who appear to hold that view. Some jurisdictions have laws that protect healthcare professionals who conscientiously refuse some medical service from legal punishment or administrative penalty [1]. These do not require that one state or defend reasons for one’s refusal; one need only state that providing the medical service in question conflicts with one’s religious beliefs or moral convictions [17]. Most—but, astonishingly, not all—require that the refusing clinician promptly refer the patient to a willing colleague. Not all such laws make exceptions for emergency situations (requiring that medical services be provided if failure to do so puts the patient’s life at risk), but even those that have emergency clauses are too vague to provide patients with suitable protection. A healthcare worker could not be legally compelled, for instance, to be vaccinated (or vaccinate others), train medical students in procedures one finds objectionable, prepare or clean instruments used in such a procedure, drive an ambulance taking a patient to another facility that provides a medical service being refused here, inform patients about their full range of medical options, schedule or submit bills for procedures, and so on.

It should be noted that these laws are not aligned with other portions of employment law [36]. Labor law sees healthcare as a profession one joins voluntarily; one also chooses where to practice. These factors greatly weaken the claim to a legal right to refuse; in the eyes of the law, if you objected to providing certain medical services, you should have made different choices. Conscientious objection would be legally permitted only for things you are compelled to do (typically compulsory military service). Employees are legally obligated to abide by employers’ lawful instructions and can be penalized if they do not. Furthermore, if someone states in advance that they intend to refuse to perform certain tasks, that is legitimate grounds for not hiring that person (or for firing someone already employed). The employer may choose to reassign this employee but is not legally obligated to do so.

There may a *moral* right to refuse, but this does not automatically translate into solid grounds for a corresponding *legal* right. Showing that a kind of action is morally right (or wrong) in some instances is not sufficient for showing that it ought to be legal (or illegal) [36]. The moral status could morally justify civil disobedience, but civil disobedience is definitionally against the law. Showing that a law should be changed does not exempt one from that law while it is in force.

#### 3.2.2. Refusal and Conscientious Commitment

Those who give priority to accommodating conscientious refusals would seem to be committing themselves to accommodating conscientious commitment as well. Conscientious commitment is the conscience-driven provision of a medical service despite being contrary to law or institutional policy.

*Case: assisted suicide*. A patient’s cancer has reached the point that treatment is no longer effective, and death is inevitable. Despite assurances that hospice would do everything possible to minimize suffering, the patient asks for the physician’s assistance in committing suicide (which is legal in that jurisdiction). The physician agrees to provide this assistance, following all the legal requirements, despite knowing that doing so is contrary to the policy of her Catholic-owned hospital. She was fired for violating policy before she could begin that process. The hospital’s policy also prohibits referring patients to other providers for assisted suicide [37].

In this case, the medical service being conscientiously provided was legal, within the standards of medical practice, and requested by the patient. Conscientious commitment can also involve providing medical services that are illegal; think, for instance, of Timothy Quill helping “Diane” commit suicide before this was permitted [38].

Sometimes conscience has led healthcare professionals to provide life-prolonging medical services because the patient had clearly refused them (typically in an advance directive) or because the treatments would be burdensome without any offsetting benefit. These typically involve ventilator support, artificial nutrition and hydration, the use of antibiotics, or attempts to resuscitate.

*Case: PEG tube for a patient with advanced dementia*. An 82-year-old female with advanced dementia falls at home (where she lives with her daughter and her family) while trying to get out of bed. She is brought to the emergency department with fractures of her hip and wrist, and a subdural hematoma. Prior to this hospitalization, there had already been difficulties in getting her to eat meals, and her physician is concerned that, with her new injuries, oral feeding will no longer be sufficient. When discussing this with the patient’s family, the daughter (the patient’s legally authorized representative) points out that her mother has made clear in her advance directive (made when her Alzheimer’s was first diagnosed) that she does not want any measures to prolong her life. She specifically refuses artificial nutrition and hydration, resuscitation, and antibiotics. The doctor, however, believes that all life is precious, regardless of its quality, and feels morally obligated to prevent this patient from “starving to death”. He orders a PEG tube to be placed.

Accommodating all conscience claims is incompatible with having a smoothly functioning healthcare system. It would treat laws, policies, and patients’ choices as though they were items in a cafeteria where you only pick the ones you want.

### 3.3. Require Compelling Reasons

What is our starting point when considering a conscientious refusal? Must the claimant provide a justification and convince others that this refusal is well grounded before it will be accommodated, or should refusals be honored unless there is a compelling objection? In other words, are conscience claims innocent until proven guilty, or guilty until proven innocent [2]? Given the power imbalance between healthcare provider and patient and the potential that the refusal would cause harm and disruption, the most common approach is to put the burden of justification on the claimant. A healthcare professional’s conscientious refusal can be disruptive and harmful, so it requires moral justification. Any healthcare provider who refuses to do what society, their employer, and their patient expect them to do owes us more of an explanation than saying merely, “I believe this is wrong”.

An objection to this approach is that, by requiring conscientious objectors to explain and defend their reasons, we are treating them as criminals. Symons, for instance, says that no such defense of an objector’s reasons is required; instead, the objector’s colleagues or institution would have the burden of showing that accommodating the refusal would cause undue harm to the patient [28]. This cannot be correct, though. It disregards the significance of professional responsibilities and places an unreasonable burden on vulnerable patients.

When someone provides reasons for a refusal, how do we decide whether they are good enough to warrant accommodation? The first step would be to rule out reasons that are clearly unacceptable. We should not accommodate refusals based on some characteristic of the patient such as race, religion, gender identity, or immigration status; these are inherently biased and violate our commitment to tolerance and justice [39]. Also, there should be a limit to how much harm, risk, or burden resulting from the refusal that we are willing to tolerate. This is not to say that *no* burden or delay is acceptable—after all, the clinician’s moral distress and autonomy are entitled to consideration—but at some point, these will become unacceptable. These judgments are difficult but not impossible. We already make somewhat similar ones when deciding whether to breach a patient’s privacy in order to protect someone else.

Hardt points out that we should be particularly careful to accommodate refusals based on the “proper ends of medicine” [40]. A healthcare provider’s refusal should be taken especially seriously if it is to avoid something incompatible with healing, relieving suffering, or other core goals of the practice of medicine. Individual refusals will need detailed analysis, and this will require a clearer understanding of medicine’s goals.

When examining the reasons people have for refusing to provide a medical service, we want to distinguish between those arising from an idiosyncratic moral view from those rooted in the widely shared moral principles that are the underpinnings of healthcare. One can imagine, for instance, a nurse refusing to administer antibiotics because of a moral conviction that bacteria have a right not to be killed [41]. We also want to rule out objections that are based on factual errors, such as the belief that vaccines cause autism [20]. It is less clear how we should handle objections based on views that are implausible but not demonstrably false. How should we establish appropriate standards of evidence, and who has the burden of proof [2]? How should we respond, for instance, to someone who defends a conscientious refusal by saying, “My religious community earnestly believes this action is immoral, and I accept their testimony” [42]?

The reasons for many conscientious refusals arise from religious conviction or deeply subjective factors that one might be reluctant to expose to critical public examination [2]. However, if the purpose of accommodating conscientious objections is to provide clinicians with “moral space” in which to use their own moral judgment, then requiring that they convince others that their judgments are reasonable seems unduly restrictive; it amounts to allowing space only for those whose judgments are similar to our own [43].

Our moral judgments are not infallible, so we ought to be open to the possibility that others’ judgments about a particular case may be superior to our own. Sulmasy argues that we ought, then, to tolerate others’ moral views—and take seriously conscientious refusals based on them—unless those views would harm what philosopher John Locke called the “common good” or be “destructive of society” [44].

Some suggest requiring that a conscientious refusal be based upon a moral principle that is deeply and genuinely held [20]. The purpose of this requirement would be to rule out cases in which someone uses a conscience claim merely as a way of avoiding an unpleasant task. A genuineness requirement would also help show that requiring the clinician to act contrarily to conscience in this case would result in significant harm to moral integrity. One way of testing the genuineness of a conscience claim would be to see whether the claimant is prepared to bear some of the burden associated with the refusal [45,46]. If you are objecting in order to protect yourself from moral taint, then you should be willing to accept the risks and consequences of doing so. Ensuring that clinicians share the costs and burdens of their refusals would also help prevent conscience claims from being used to mask bias, dislike for the patient, or a desire to avoid onerous work. A healthcare professional who is willing to accept the costs associated with refusals will be more likely to be making claims that are sincere and that involve moral principles that are deeply significant for them.

### 3.4. Require Referrals

In trying to balance the demands of conscience and the needs of patients, the usual compromise (found in many laws and policies) requires that the healthcare provider who refuses to provide a particular medical service must promptly and smoothly transfer that patient to a colleague who is willing to do it [47,48,49,50]. Such referrals are said to minimize the delays and burdens caused by refusals and limiting the power of healthcare providers to impose their moral views on their patients [51].

If a healthcare professional is willing to make smooth, prompt transfers of care when they conscientiously refuse to provide some medical service, there is no need to ask the person refusing to explain or defend that moral judgment. The refusal might be based on factual error, bias, or solid moral insight—we just do not care if you are willing to step out of the way and let others get on with it. Referral allows us to avoid taking sides or getting into debates about deep moral disagreements.

The referral requirement functions as though we assume that the person objecting is making a moral mistake. If we shared the objector’s concerns, we would join in trying to prevent this sort of action; we would work at changing laws, policies, and standards of practice. If we thought the objector might have a good reason for refusing to provide this service, we would examine those reasons before deciding how to proceed. But the refusal requirement does none of that; it just brushes aside the objection by moving the patient elsewhere.

This is troubling because, as history illustrates, the medical profession has sometimes accepted practices that were seriously immoral; in such cases, referral could be morally wrong, too. The question of which conscientious refusals are permissible (or obligatory) is different from the question of which should be accommodated. It may be appropriate to accommodate some refusals that are ethically unwarranted, and accommodation will involve referral.

Understandably, clinicians asked to make such referrals think doing so constitutes facilitating a serious wrong, thus making them share moral responsibility for it. This has greater weight if the referral requirement includes a clause requiring that the objector provide the care in question if the situation is an emergency and no willing colleague can be found promptly. Some ethicists argue that this complicity is reduced to a level they should find tolerable particularly if, in the process of making the referral, they make their objections clear and have no intent to cause harm [52].

Symons [28] is among those who are not convinced; he argues that referrals still constitute direct participation in wrongdoing and no objecting clinician should be compelled to act contrary to conscience. At most, he says, patients could be directed toward publicly available information about other providers without any attempt to transfer their care to any individual. He acknowledges that this will increase burdens for patients and colleagues but maintains that these are not substantial enough to warrant violating a healthcare professional’s conscience.

A direct referral requires the healthcare professional who is refusing to provide a service to identify a willing colleague and take steps to transfer the patient’s care. If the care at issue takes place, the referring clinician plays a role in facilitating it. That role can be reduced by having the patient take over some of the steps in this transfer. The refusing clinician could simply provide a list of names and contact information for others who are likely to be willing to provide such care. The clinician would not contact or recommend anyone on that list; the patient would be responsible for making the calls, scheduling appointments, and so forth.

This would seem to reduce the refusing clinician’s complicity in the contested service to the point that it should be tolerable to them, but they would probably still object; they may see it as equivalent to saying, “I don’t kill people, but here’s a list of assassins I found on the internet”. An alternative would be for the information about alternative healthcare providers to be supplied via the institution, rather than the refusing clinician [53]. Either way, this would also be a major barrier for patients, who are often already struggling to navigate a complex system while enduring medical problems. That burden requires a justification, and that seems to lead us back to requiring and evaluating the clinician’s reasons for refusing.

Conscience’s judgments about right and wrong (and our associated experiences of satisfaction or guilt) are also associated with what other people do if we regard ourselves as being involved in those actions. Whether you are complicit in and morally accountable for others’ actions depends upon several factors: Do you share the intentions of the person performing the action? What is your role in that action, and would the action still have taken place without your involvement? Were you obligated (morally, legally, or professionally) to participate? Were you compelled to participate against your will [39]?

### 3.5. Match Patients with Providers

Another strategy for dealing with conscientious refusals focuses on reducing the number of refusals we have to deal with. These refusals arise when the treatment the patient chooses falls outside the boundary of what the healthcare provider finds morally acceptable. We could avoid refusals, then, by matching patients with healthcare providers who share the same fundamental values and moral standards. The medical services the clinician would refuse to provide are ones that those patients would not want to choose. We tend to trust people more when we know that we have shared values, so this matching approach could help build strong patient–provider relationships.

One proposal is to have medical licensing boards keep records of healthcare professionals’ moral values and a list of any medical services they refuse to provide [54]. Boards would then make this information public so that patients could select someone whose values match their own. If a patient requests a treatment the clinician conscientiously refuses to provide, there would be no requirement to make a direct referral; the institution could provide the patient with a list of others who might be willing to provide the service.

There are a number of practical concerns about this matching strategy. There would not be time to consult these lists in emergency situations. It would not account for all of the people on the healthcare teams involved in one’s treatment, or for shift changes, or for the limitations of insurance networks, finding moral diversity in rural or culturally homogeneous areas, and so forth. McLeod argues that it would also undermine society’s trust in the medical professions by signaling that they would put their own personal views ahead of the patient’s needs [55].

### 3.6. Summary

All the strategies that have been proposed for responding to conscientious refusals face challenges, but the extreme positions—either overriding all refusals or accommodating them all—should be rejected. The best approach will involve both examining the reasons for the refusal and typically requiring prompt referrals. Matching patients with healthcare providers who share their fundamental values could play some role in reducing the frequency of refusals, but it is not suitable for addressing the refusals that do arise.

We can reach a deeper understanding of the need for examining reasons and making referrals by looking more closely at the social and moral obligations to provide healthcare, and at the nature of conscience.

## 4. Obligations to Provide Care

### 4.1. Fiduciary Responsibilities

Healthcare professionals are gatekeepers controlling patient access to medical care; they must play this role in accordance with fiduciary responsibility [55]. A fiduciary is a professional who acts on behalf of a patient. It is a position of trust founded on the principle that the client’s interests take priority over those of the fiduciary. The ethical analysis of conscientious refusals is not, McLeod argues, simply a matter of balancing healthcare providers’ interests against those of patients. Instead, the fiduciary nature of the clinician-patient relationship requires that the latter’s interests be granted priority. From this starting point, she argues that the typical compromise proposals (requiring that objection clinicians promptly refer patients to a willing provider) are unacceptable because of the harms and burdens they involve for patients; they fail to put patients’ interests first.

In addition to the fiduciary duty to patients, healthcare professionals may also have a quasi-fiduciary duty to society [55]. It is a moral obligation to ensure that medical services are provided competently and consistently; this, too, is said to be violated when we accommodate conscientious refusals. She argues that we can value conscience without accommodating refusals by allowing them to express their concerns, engage in public discussion, and try to convince us to change standards of care. But this misses the point that one may not wish to impose one’s moral or religious principles on others or convince others to agree with you; instead, you may simply wish to avoid doing what you believe is wrong. Nor would you like to present your views publicly to a critical audience.

### 4.2. Professional Roles

Professionals have power in their interactions with patients, and there are legitimate social and professional expectations about how this will be used [56]. For example, public defenders have a professional obligation to defend even people they find utterly appalling, people they would otherwise absolutely refuse to support. When the rest of us see them engaged in these tasks, it is clear to us that this is done as a professional duty and that it is separate from personal morality. To have a society that is just, we must accept that personal morality will sometimes be overridden by professional obligation. Zolf puts this rather well: [57] (p. 148)

“…the purpose of a profession is to provide a service in a manner that is, at least to some extent, standardized and consistent. This enjoins professionals with a set of responsibilities and expectations. Medical professionals pass rigorous training and licensing processes, and pledge an oath to use that training for the good of their patients. If members of a profession can opt out of the standards that define it, creating exceptions to their responsibilities and the expectations associated with them, those exceptions must conform to some standard of reasonability in order not to jeopardize the delivery of the profession’s associated service. Allowing professionals to exempt themselves from these responsibilities entirely at will is inconsistent with the objective of standardized, consistent delivery, and at odds with the purpose and concept of a profession”.

The moral expectations associated with a professional role do not always match the personal moral compass of the person in that role. It is unrealistic for a professional to expect or demand that their role’s moral expectations will always cohere with their own sense of moral rightness [56]. When you join a profession, you do so voluntarily, and when you take on a professional role, you automatically take on a network of responsibility and accountability. This involves patients, colleagues, institutions, third-party payers, and society more broadly. As Card put it, “We may exercise conscientious objection to involvement in certain activities but surely we cannot entirely float above the network of obligations in which we have immersed ourselves” [56] (p. 122).

To ethically fulfill a role, one must sometimes act passively and obediently; this was pointed out by Immanuel Kant [58,59]. For instance, soldiers must be obedient even if they disagree with strategy. They may refuse to obey an unlawful order, but this is far more restrictive than allowing them to disobey any order that conflicts with their conscience. Similarly, citizens may not withhold taxes because of disagreements with public policy, but this does not make them morally accountable for those policies. By the same reasoning, healthcare providers are hired to provide care competently and reliably, not to make their own policy decisions or take over the patient’s entitlement to guide their own care. People who occupy a particular professional or public role are not permitted to substitute their own personal moral views for the standards and obligations inherent in those roles. In that role, they are limited to the moral principles that are internal to it. For example, lawyers must stick to their roles even if they believe that the client they are defending is guilty or that the law being applied is unjust [60]. We expect healthcare professionals to put patients’ needs above their own personal views and interests [55]. Being a physician is a “standing social signal” that one has committed to doing this [61] (p. 76).

One drawback of inhabiting a professional role is that it can slip into thinking of oneself as a “cog in a machine” without moral agency or responsibility, unable to change anything about the system in which one works. This is part of our larger tendency to avoid distressing moral issues [32]. When a patient’s care causes moral distress, we may try to reduce our contact with them; nurses might do this by switching shifts, while doctors might “turf” the patient to another unit or provider. We may try to reframe the issue in a less troubling way, perhaps making it about cost containment, risk management, or regulatory compliance. Most commonly, we try to “kick the can down the road”, putting off emotional conversations and hard decisions. But when conscience speaks to us, we must listen; its pronouncements are not always correct, but they must be examined, and we must take responsibility for how we respond.

These medical roles are not a morality-free zone where anything goes as long as it is part of the job description. There is a moral core here, and it is found in the values central to the practice of medicine—not in the conscience of the individual clinician. Providing essential healthcare to preserve or restore health, to relieve suffering, to help people adapt to changes in their bodies and lives—these are among the core goals of medicine [62]. Society grants healthcare professionals a sort of monopoly on medical treatment on the understanding that they will give priority to patient care [63]. Conscientious refusals undermine this by substituting an individual’s goals for those inherent to the profession.

Not all ethically wrong actions can be made permissible by performing them as part of a professional role [56]. Professionals must also reflect upon the legitimacy of that role and the demands it makes. Medical history presents us with too many instances of clinicians engaging in practices we now regard with horror: the Nazi program of murdering people with cognitive disabilities, involuntary sterilization, and so on [64]. But there is also danger in professionals adopting this “big picture” approach too readily as a means of avoiding their professional responsibilities. What we need (as in so many aspects of life) is a proper balance. To find that balance, we will need a clearer understanding of conscience.

## 5. Conscience

We experience conscience chiefly as an emotion-laden moral evaluation. We may feel guilt or satisfaction regarding our past actions or when we anticipate future actions; the latter arises primarily in cases of conscientious refusal. These emotions serve as motivation, pushing us to do what would bring moral satisfaction and avoid what would cause guilt. Although conscience is often seen as a source of moral knowledge, the mechanism for that is unclear [46]. If it were, in fact, a mechanism for determining moral rightness and wrongness, we would expect greater agreement about ethical issues. Religious traditions hold that one’s conscience is a channel of moral communication from a divinity [65], but conscience is not merely a religious concept, and its judgments are not shielded from critical examination [40]. It is generally agreed that our judgments of conscience are understood to be the result of the moral standards we have internalized from our culture, peers, education, and so forth. What is important here is not whether the act in question is actually morally wrong but that one sincerely believes (on the basis of conscience) that it is [66].

Failing to act in accordance with one’s conscience results in feelings of guilt; the judgments of conscience are powerful drivers of our behavior because we try to act morally and avoid guilt. If you violate a principle that is particularly deeply held, if it is a deep part of who you are, then the violation harms your moral integrity; it is a sort of self-betrayal. Sulmasy points out, however, that it is impossible to avoid all guilt [39]. We are flawed creatures trying to deal with a complex, fast-moving world; anyone who feels no guilt at all must have a seriously defective conscience. But bear in mind that not all violations of conscience are equally serious, and not all are equally entitled to accommodation by others [2]. And often, conscience is violated during attempts to uphold some competing moral commitment.

Conscience is fallible. The fact that a moral judgment is felt strongly is no guarantee that it is correct. Our conscience is constructed from the ethical standards held in our culture, seen in the judgments made by our parents and colleagues, taught to us in classrooms, depicted in entertainment, and so on. The philosopher Immanuel Kant calls this *artificial conscience* and warns against relying on the judgments that arise from it [46,59]. For instance, Hannah Arendt describes Adolf Eichmann, a bureaucrat responsible for putting into action the Nazi’s genocidal “final solution”, as someone who thought of himself as a moral, dependable, upstanding person [67]. He felt guilt—not for facilitating the murder of millions of innocent people, but for instances in which he had allowed Jews to escape. He had internalized and incorporated into his conscience the hatred and bigotry of his culture and came to regard it as a solid moral foundation. The philosopher John Stuart Mill pointed out that, although we recognize the fallibility of others’ judgments and reasoning and are always on the lookout for their mistakes, we experience difficulty in accepting that we are similarly fallible [34].

To be morally responsible people, we must examine our moral views rationally and intersubjectively build a solid moral framework capable of producing moral judgments that merit serious consideration. This process changes artificial conscience into what Kant terms *critical conscience*. When someone claims that their conscience will not allow them to provide a certain medical service, Kant would say that we need to examine this person’s understanding of morality in order to determine how much weight to give their view. Kennett suggests that Kant’s view regarding freedom of conscience “… is not the freedom to deny service or to demand accommodation from others; it is the freedom and indeed the obligation to argue one’s views in the public arena or in discussion with fellow professionals …” [46] (p. 74).

Even when one’s conscience is applying proper ethical principles, it can do so too strictly or too permissively [39]. We may be too ready to allow self-serving exceptions from moral standards or too readily accept weak reasons for breaching them. On the other hand, we may give too little weight to moral reasons against a judgment arising from conscience or insist upon an unreasonable degree of moral purity. This is yet another reason that conscience requires thoughtful analysis and caution.

*Case: refusal to lie*. An unmarried teen girl was brought to a hospital in Saudi Arabia for a spinal issue, but her American doctors discovered that she was pregnant as well. Two of her doctors arranged, with the girl’s consent, to have her transferred to a hospital in another country where she would receive both spinal treatment and an abortion. They agreed that her parents would be told only about the former. A third doctor involved in her care refused to participate in lying to the parents and disclosed the abortion to them. The girl was killed by her family for bringing dishonor upon them [68].

Flawed and fallible it may be, but conscience must nevertheless be taken seriously. Every moral judgment, every deliberated choice, is rooted in conscience; therefore, concludes Curlin, “… acting conscientiously is the most fundamental of all moral obligations” [8] (p. 31). Our response to flawed judgments of conscience should be ethical analysis and debate, not the dismissal of conscience altogether. Our tolerance for moral views we do not share is mutual respect for conscience, a way of showing respect and maintaining connections with others. As always, societies (and institutions) must try to find the appropriate balance between tolerating too much and too little.

## 6. Recommendations

We recommend that we avoid conscientious refusals when we can, think carefully about whether to refuse, and take responsibility for our refusals. These recommendations are summarized in Table 2.

First, we should take steps to avoid situations in which healthcare would normally be expected to provide services that they would refuse. One step in this direction is these providers choosing their medical area and scope of practice to avoid areas they find morally objectionable [2]. Publicly make clear any services you will not provide or any categories of patients you will not serve. You or your institution should make available information about other providers who would be willing to provide those services. Even here, though, there are limits on what should be accepted. Outright bias should not be tolerated. A urologist in Florida was opposed to the Affordable Care and Patient Protection Act and posted this sign in his office window: “If you voted for Obama … seek urological care elsewhere. Changes to your healthcare begin right now, not in four years” [69]. There is no place for that in ethical healthcare.

The disruptions and harms that result from a clinician’s conscientious refusal have been described above, and concern about such harms is at the center of restrictions on accommodating conscientious refusals. Munthe sums it up [36] (p. 63):

“Policies making room for such refusal must not threaten the functionality of important social institutions of any sort. Although civil liberties may be valued highly, they are always subject to such constraints, as a society exists for the common good of its members, none of whom has the authority to hold the rest hostage, forcing them to bend to the “refuser’s” own personal idea of how society should be”.

Healthcare professionals who anticipate that they will conscientiously object to providing certain services should explain their reasons. These reasons should then be vetted to ensure that they are not based on bias or empirical falsehoods. The purpose of examining the reasons for refusal is to identify cases in which providing the medical service in question would be seriously wrong. That argument would have to be made using what Rawls describes as *public reasons* [70], appealing to moral principles that are internal to the practice of medicine or part of our commonly shared morality.

Which values (if any) are inherent in healthcare is a topic of ongoing debate. Philosopher Alasdair MacIntyre [71] described the practice of medicine as a coherent, complex set of human activities organized around promoting a set of shared goals and values. These are linked with a set of duties for those who pursue these goals as members of the healthcare professions and the moral virtues that would make one a good member of those professions. The pursuit of health and healing are obviously central here, as well as relieving suffering, maintaining or restoring function, and supporting people as they adapt to new ways of living with their bodies and environments. To these values, one would add fundamental moral principles from our common morality, such as distributive justice and respect for autonomy [72].

To the extent that the reasons for refusal involve religious belief or personal moral conviction, it is unlikely that they will seem compelling to anyone who does not already share them. Such views should be tolerated and accommodated, *provided that a prompt, smooth referral is made* with minimal disruption of the patient’s care. As discussed above, this involves a degree of complicity that the refusing clinician is likely to find very troubling. However, we believe that the moral obligations arising from the professional role suffice to override that concern. This referral would take place only if the refuser is unable to show that the medical service in question is seriously at odds with the values associated with healthcare. When considering the interests of the patient against those of the healthcare provider, the balance tips in favor of the patient.

A refusal can be accommodated (by requiring a prompt, smooth referral) even if we do not believe there are good moral grounds for refusing. This accommodation is part of accepting our moral and personal differences, allowing for the ongoing discussion of medical ethics. When the refuser can show that providing a medical service is seriously wrong as judged according to the values inherent in medicine and our shared morality, then no referral is required, and the situation should be investigated further before continuing with the disputed treatment.

Healthcare employers and workplaces should foster an environment in which staff are able to voice their concerns and objections and participate in constructive dialogue about how best to handle difficult situations [32]. This would be the first stage in exploring whether a conscientious refusal would be appropriate, how a refusal would affect others, and so forth.

Our accommodation of conscientious refusals will sometimes result in significant costs or burdens for patients. These may involve the costs of patient transportation, seeking care outside an insurance network, medical decline associated with delayed treatment, and so forth. When feasible, some of these costs should be borne by the refusing healthcare provider. We have an obligation to take responsibility for our actions and the effects they have on others. This approach is explored and defended by Kennett [46]. This will also reduce the frivolous or inappropriate use of conscience claims and help to show that the underlying ethical principle is deeply and genuinely held by the person refusing.

## 7. Conclusions

Because of the impact conscientious refusals have on patients and colleagues, responsible clinicians will use them sparingly [66]. We want the problems we face to be simple, with clear solutions. When we encounter a morally distressing situation in a patient’s care, we want to be able to say simply, “This is wrong—I won’t do it”. The real world, however, is far from simple. Some things are simply wrong, but far more are messy, painful admixtures of good and bad, light and dark. Rather than trying to distance ourselves from these situations by refusing to participate, we have an obligation to think harder about the ethical issues involved. Conscientious refusals have their place, but let us not let them stray too far beyond that proper place.

## Figures and Tables

**Table 1:** Strategies for responding to conscientious refusals by healthcare providers (HCPs).

Strategy	Challenges
Override all refusals	Moral distress for HCPs, risk of continuing moral wrongs
Allow all refusals	Burden on patients, “conscience creep”
Require compelling reasons	Which standards for ethics, evidence?
Require direct referral	Moral distress for HCPs, burden on colleagues, risk of continuing moral wrongs
Require indirect referral	Burden on patients
Match patient and HCP values	Practicality, does not resolve refusals that do arise

**Table 2:** Recommendations.

Prevent refusals	- Make public what you will not do - Match patients to providers when practical - Provide information about willing healthcare providers
When considering refusing	- Look for signs of bias, error, oversimplification, etc. in your moral judgment - Discuss the issue with colleagues - Take your professional obligations seriously
Refusing	- Explain your reasons, but do not lecture or accuse the patient of being immoral - Smoothly and promptly transfer the patient to a willing provider - Be prepared to take on costs or burdens arising from your refusal

## Data Availability

No new data were created or analyzed in this study. Data sharing is not applicable to this article.

## Public Involvement Statement

No public involvement took place in any aspect of this research.

## Guidelines and Standards Statement

This manuscript was drafted against the LETTERS guidelines for biomedical ethical analysis [73].
